# Supplementary material for: Phylogenomics investigation of sparids (Teleostei: Spariformes) using high-quality proteomes highlights the importance of taxon sampling
Source: Commun Biol. 2019 Nov 1;2:400. doi: 10.1038/s42003-019-0654-5 (PMC6825128; doi:10.1038/s42003-019-0654-5)
Supplement: Supplementary file 1 — Supplementary Material [file 42003_2019_654_MOESM1_ESM.pdf]

SUPPLEMENTARY FIGURES

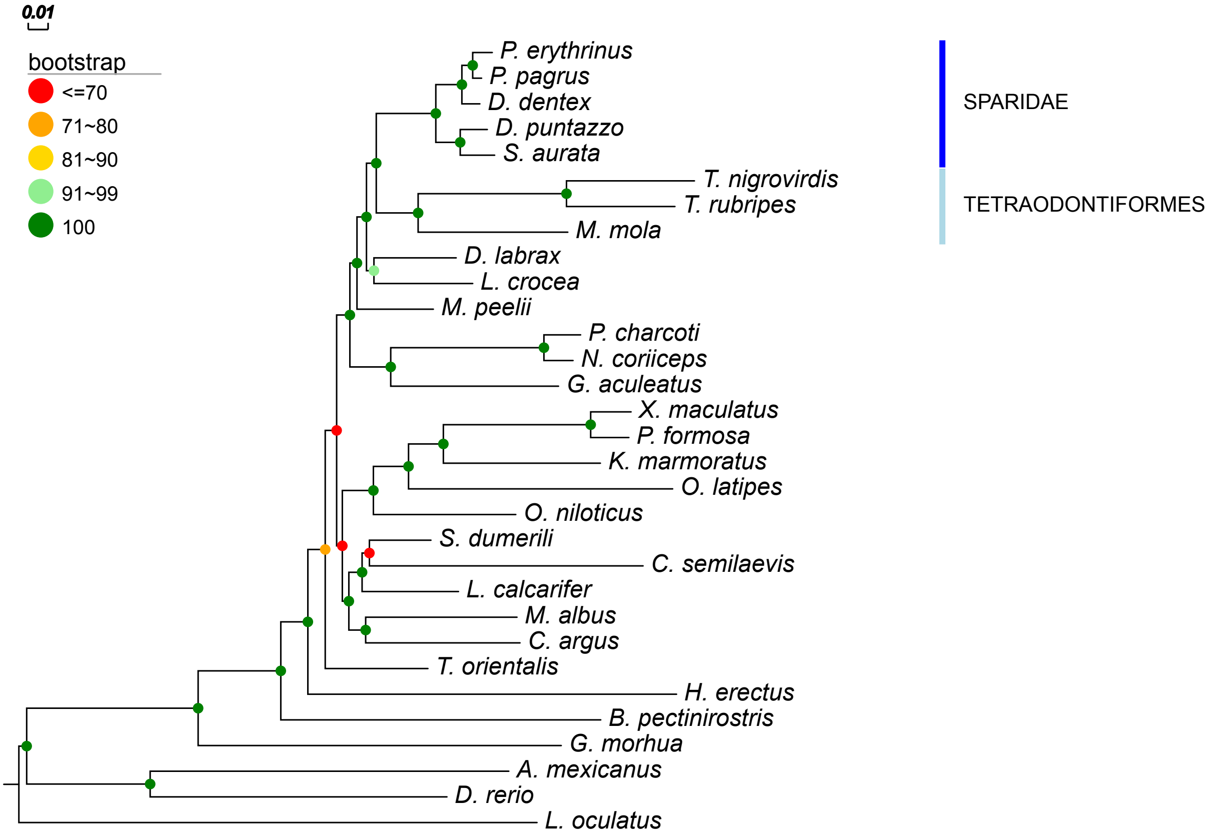

Supplementary Figure 1 Maximum likelihood tree of 533 concatenated PorthoMCL groups using RAxML

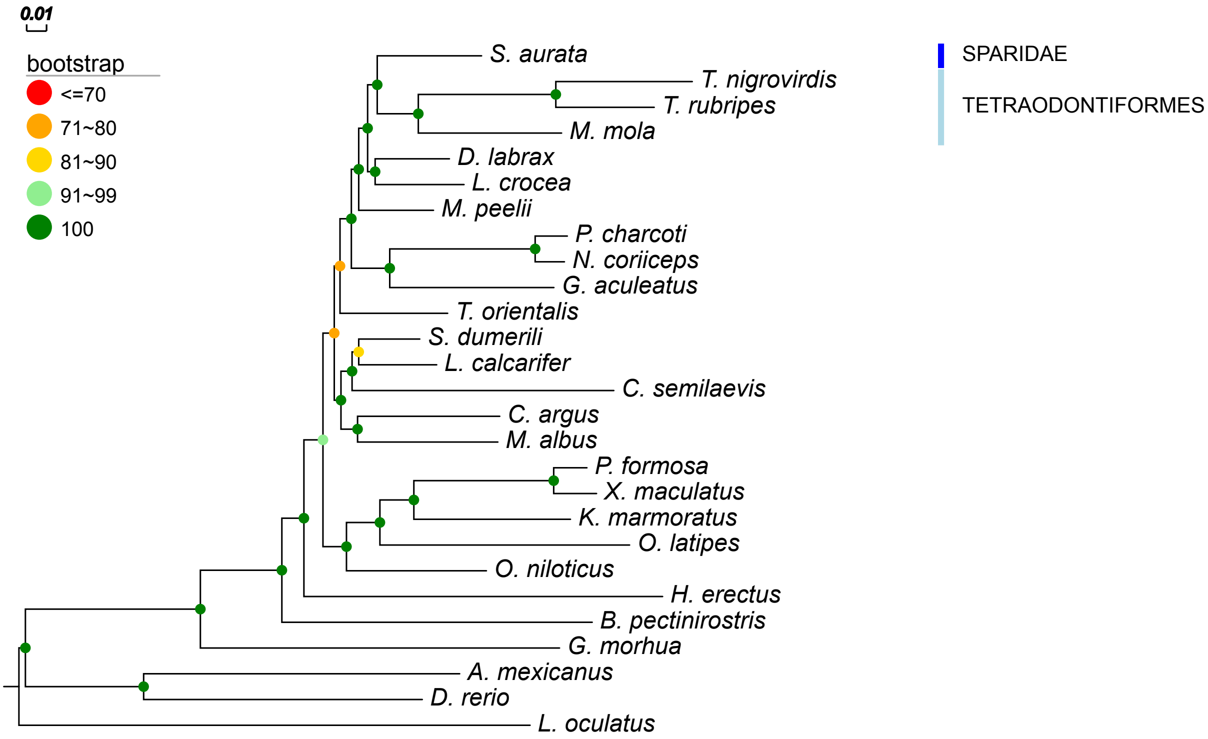

Supplementary Figure 2A Maximum likelihood trees of 793 OrthoFinder groups using different subsets of Sparidae/Tetraodontiformes species: A) only seabream from Sparidae, B) sunfish removed from Tetraodontiformes and C) only sunfish from Tetraodontiformes

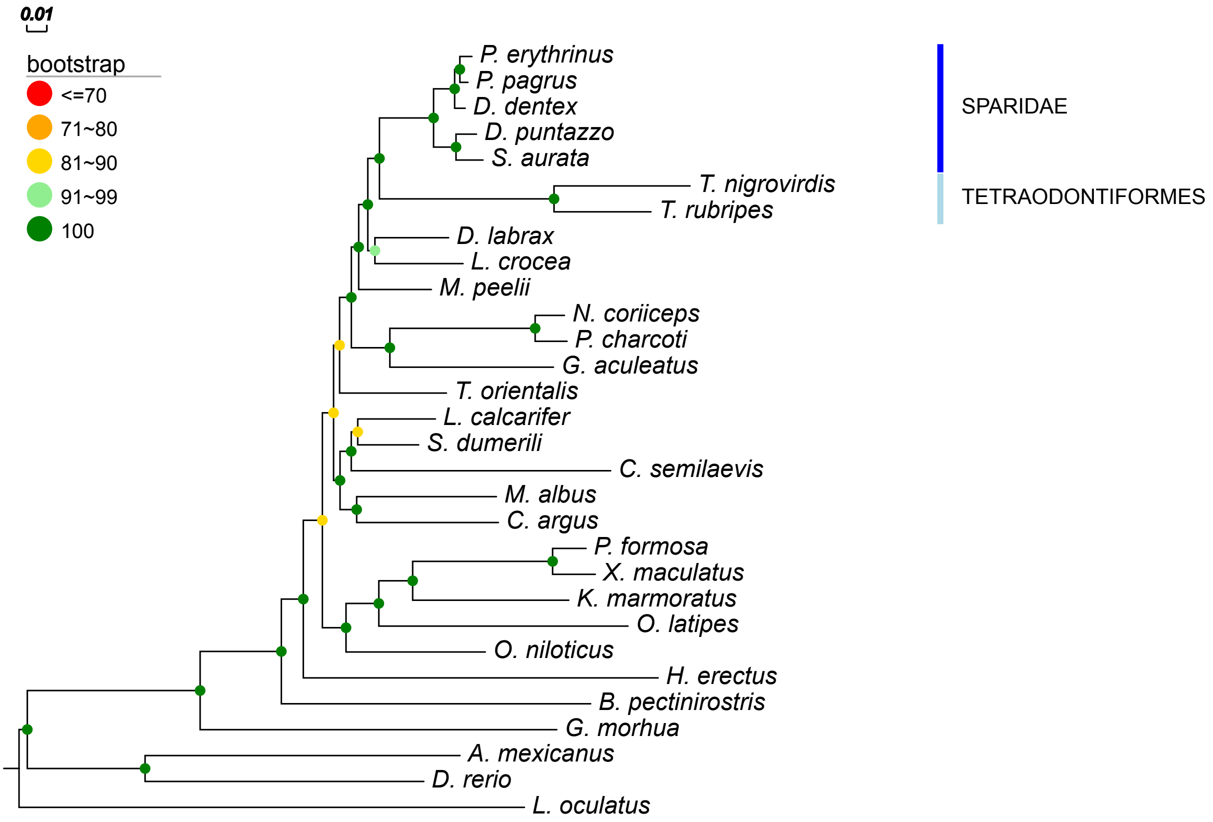

Supplementary Figure 2B Maximum likelihood trees of 793 OrthoFinder groups using different subsets of Sparidae/Tetraodontiformes species: A) only seabream from Sparidae, B) sunfish removed from Tetraodontiformes and C) only sunfish from Tetraodontiformes

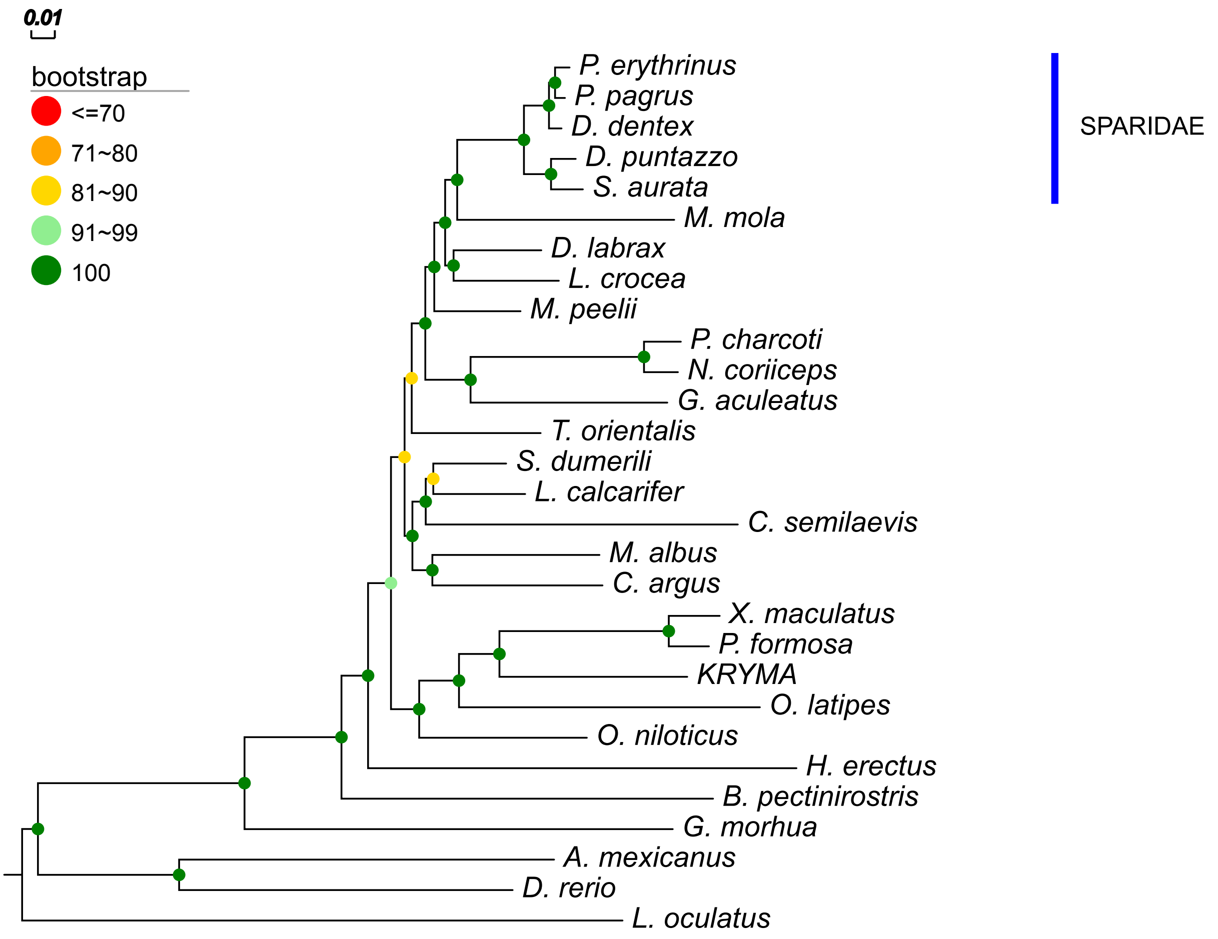

Supplementary Figure 2C Maximum likelihood trees of 793 OrthoFinder groups using different subsets of Sparidae/Tetraodontiformes species: A) only seabream from Sparidae, B) sunfish removed from Tetraodontiformes and C) only sunfish from Tetraodontiformes

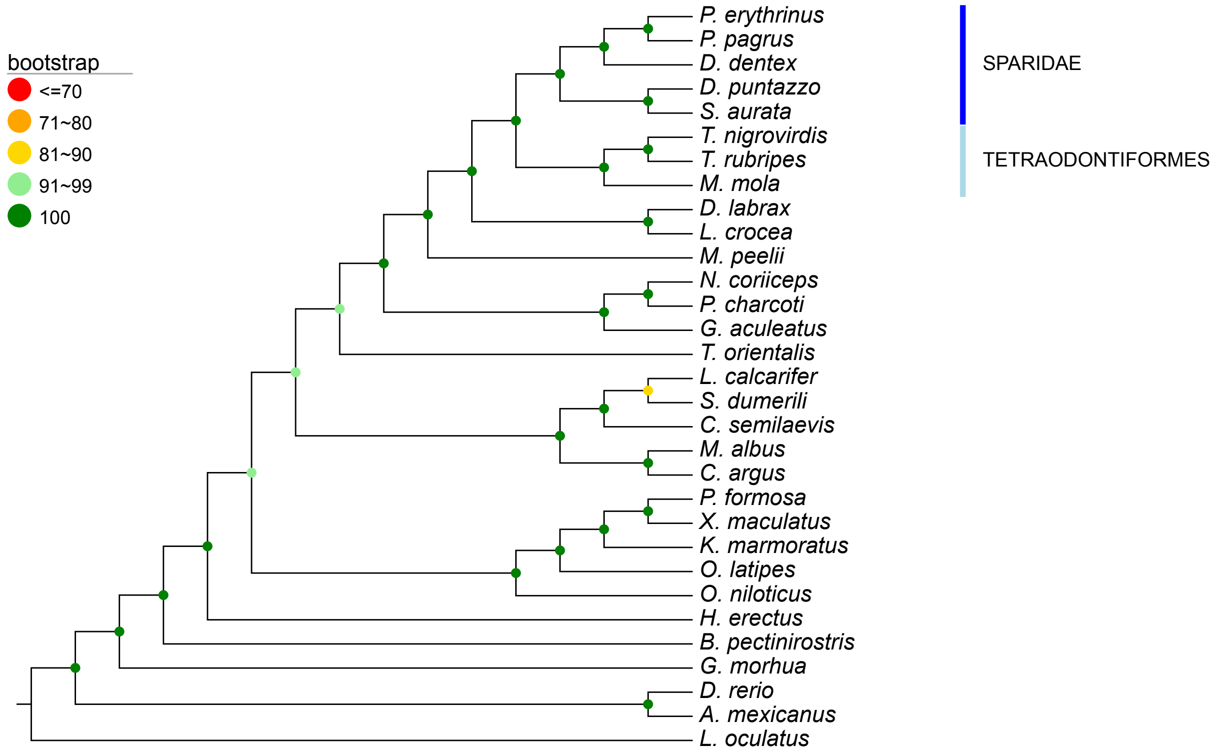

Supplementary Figure 3A Consensus trees of 100 jackknifed replicates (70% of groups kept) for: A) OrthoFinder and B) PorthoMCL groups

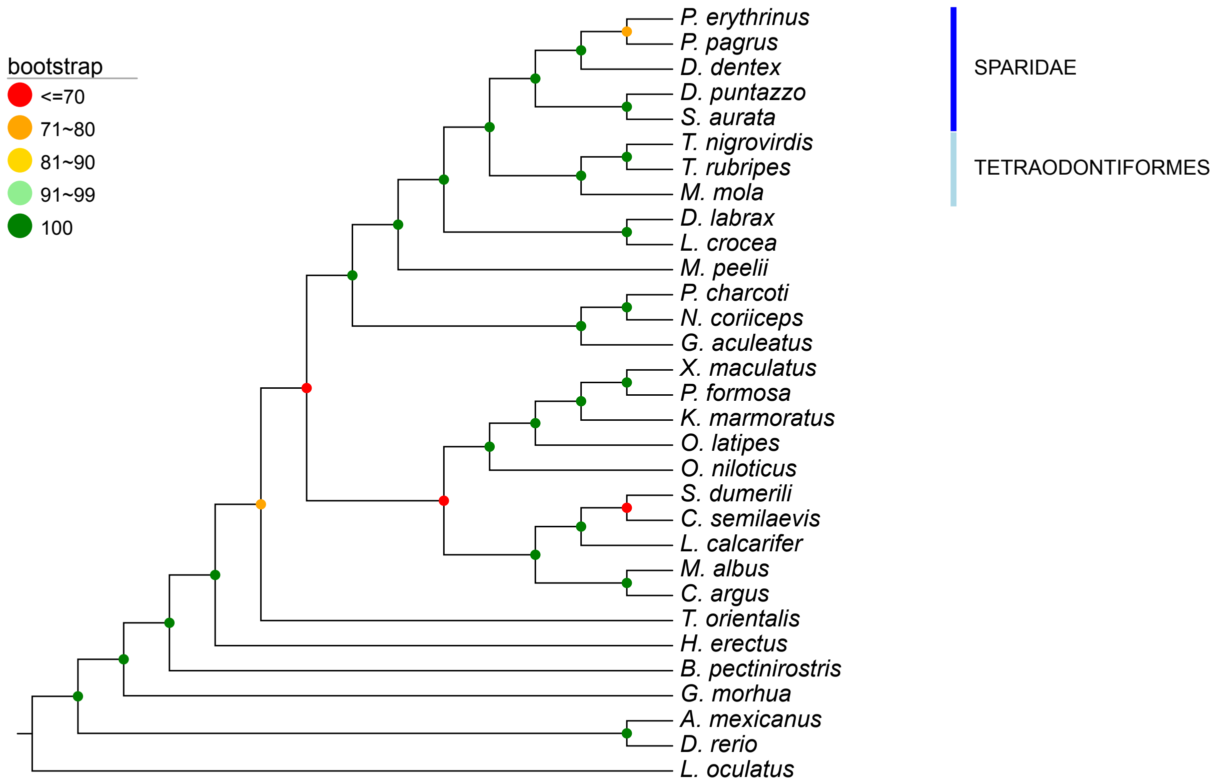

Supplementary Figure 3B Consensus trees of 100 jackknifed replicates (70% of groups kept) for: A) OrthoFinder and B) PorthoMCL groups

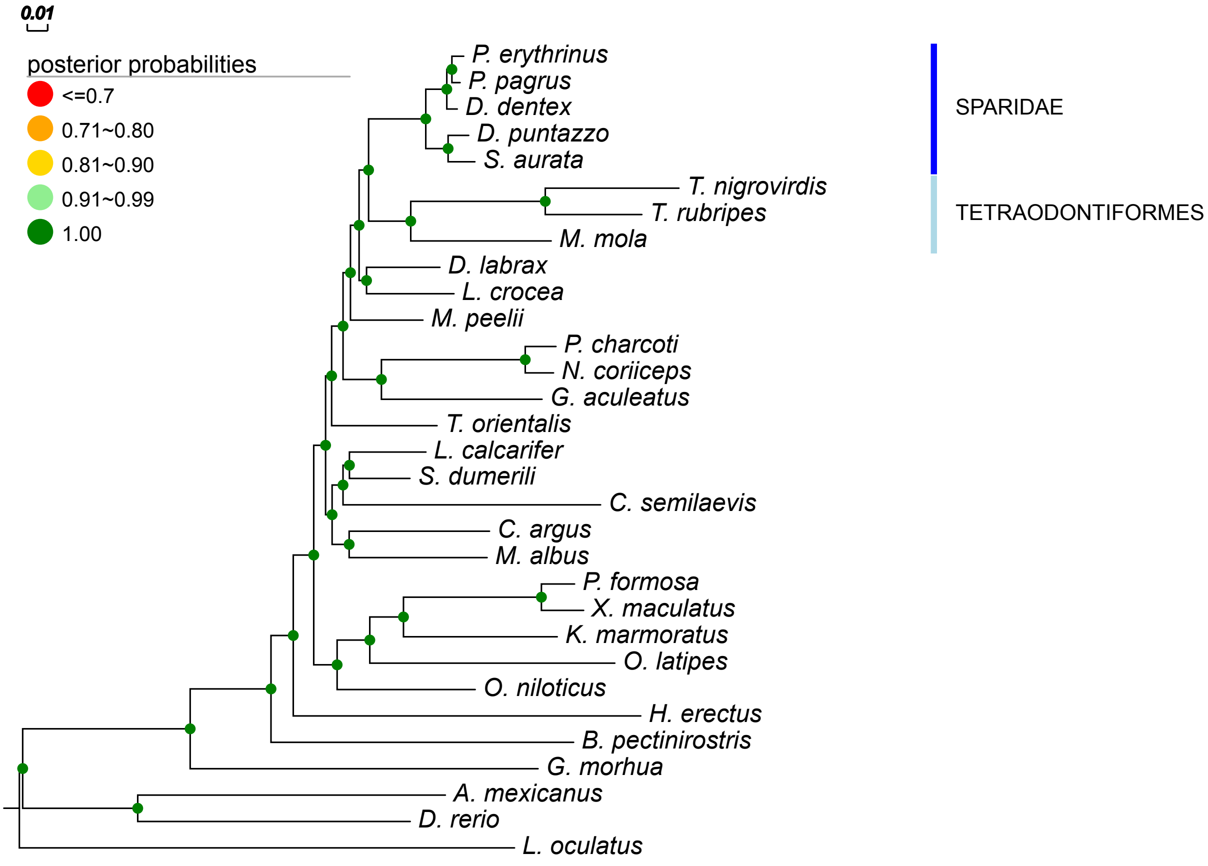

Supplementary Figure 4A Bayesian consensus trees after 25% burn-in for two parallel MCMC chains for: A) OrthoFinder and B) PorthoMCL groups

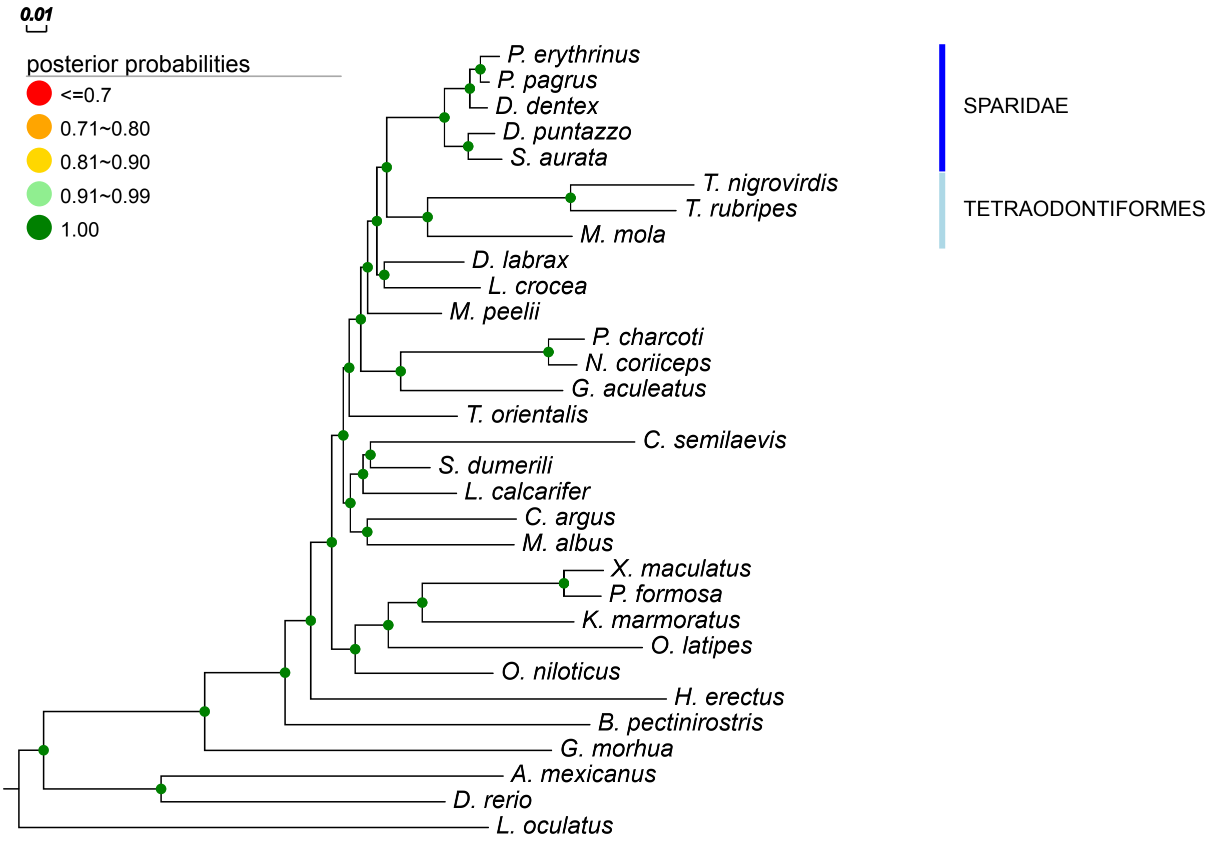

Supplementary Figure 4B Bayesian consensus trees after 25% burn-in for two parallel MCMC chains for: A) OrthoFinder and B) PorthoMCL groups

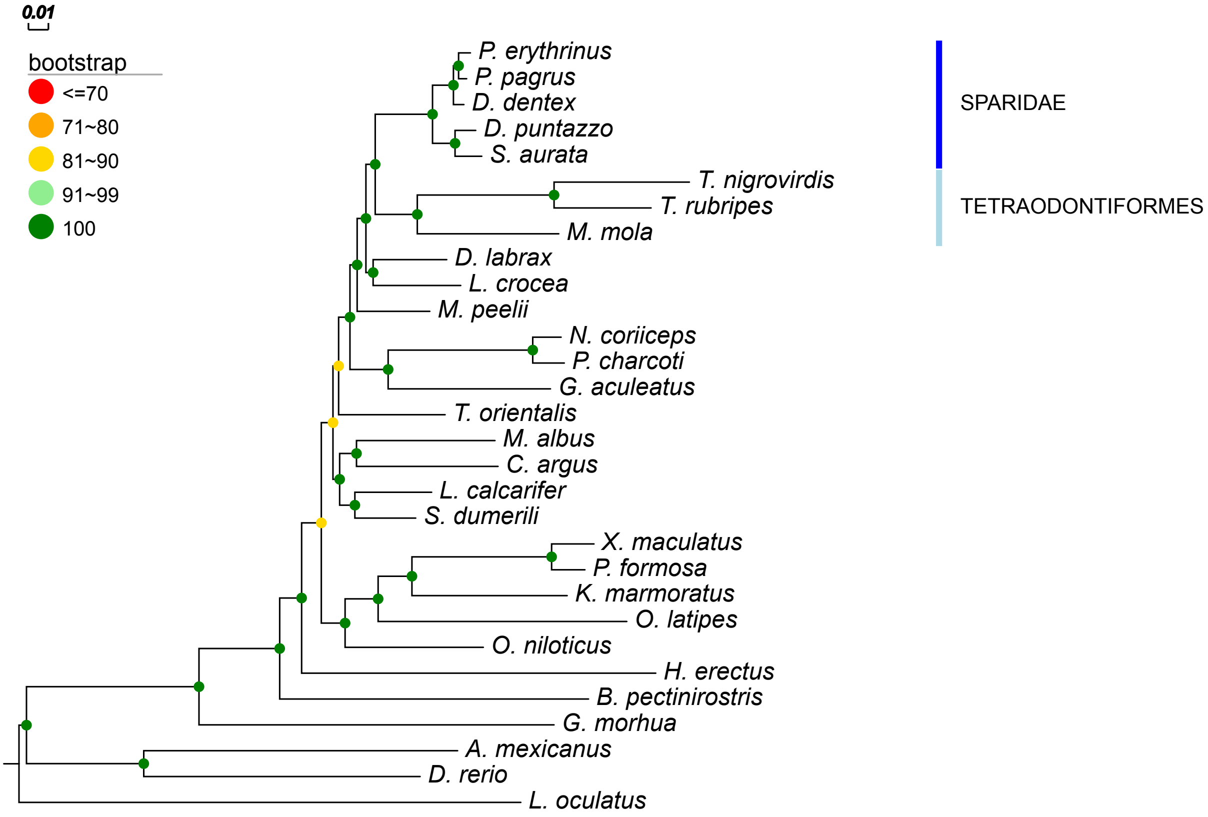

Supplementary Figure 5A Maximum likelihood tree of 793 OrthoFinder groups A) without tongue sole and B) without tongue sole and pacific bluefin tuna

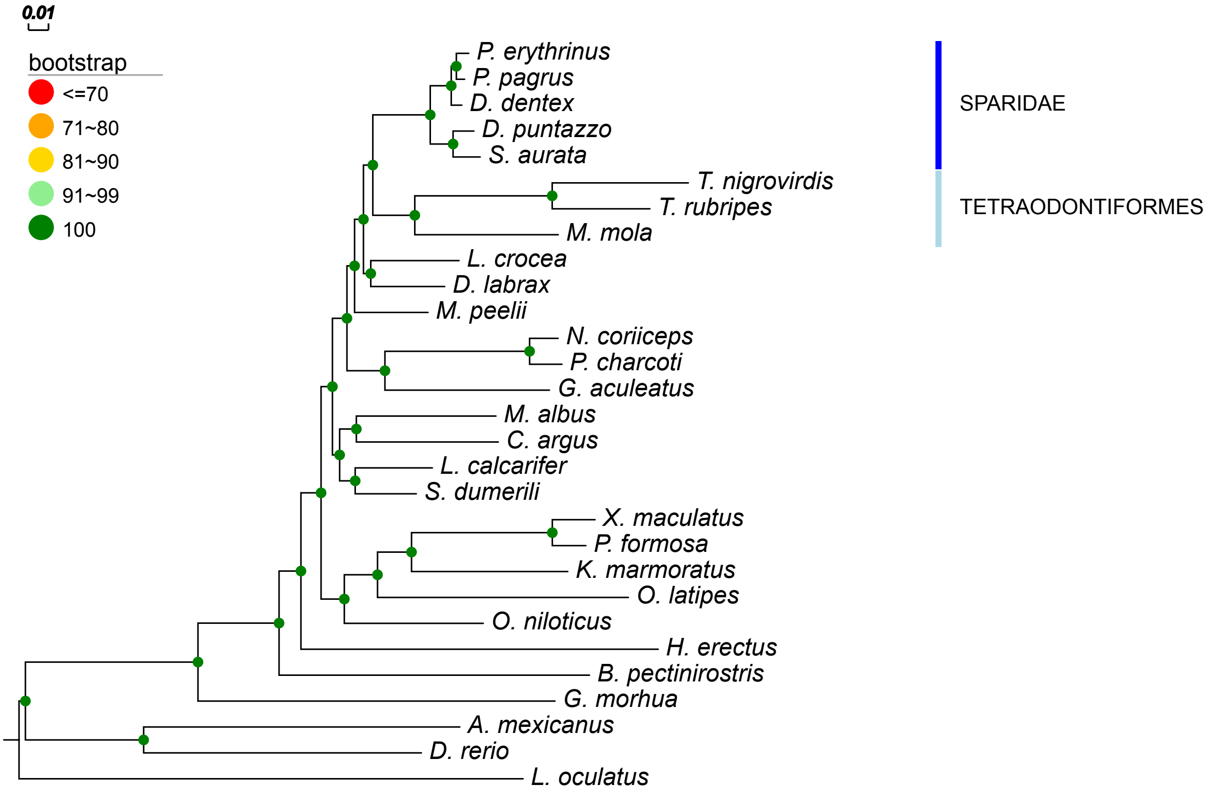

Supplementary Figure 5B Maximum likelihood tree of 793 OrthoFinder groups A) without tongue sole and B) without tongue sole and pacific bluefin tuna

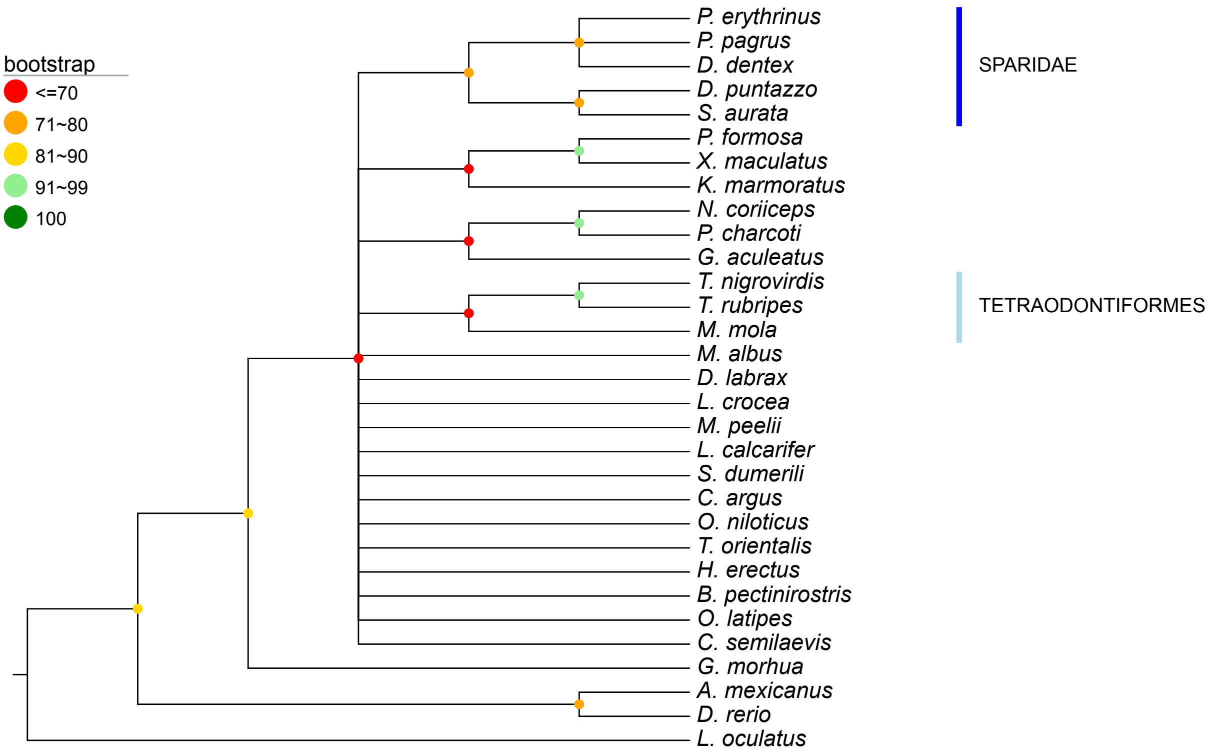

Supplementary Figure 6A Gene tree analysis consensus trees for: A) 135 OrthoFinder and B) 78 PorthoMCL groups with all 31 species present. Figures C and D show the results of IC/ICA calculation by RAxML, for OrthoFinder and PorthoMCL respectively

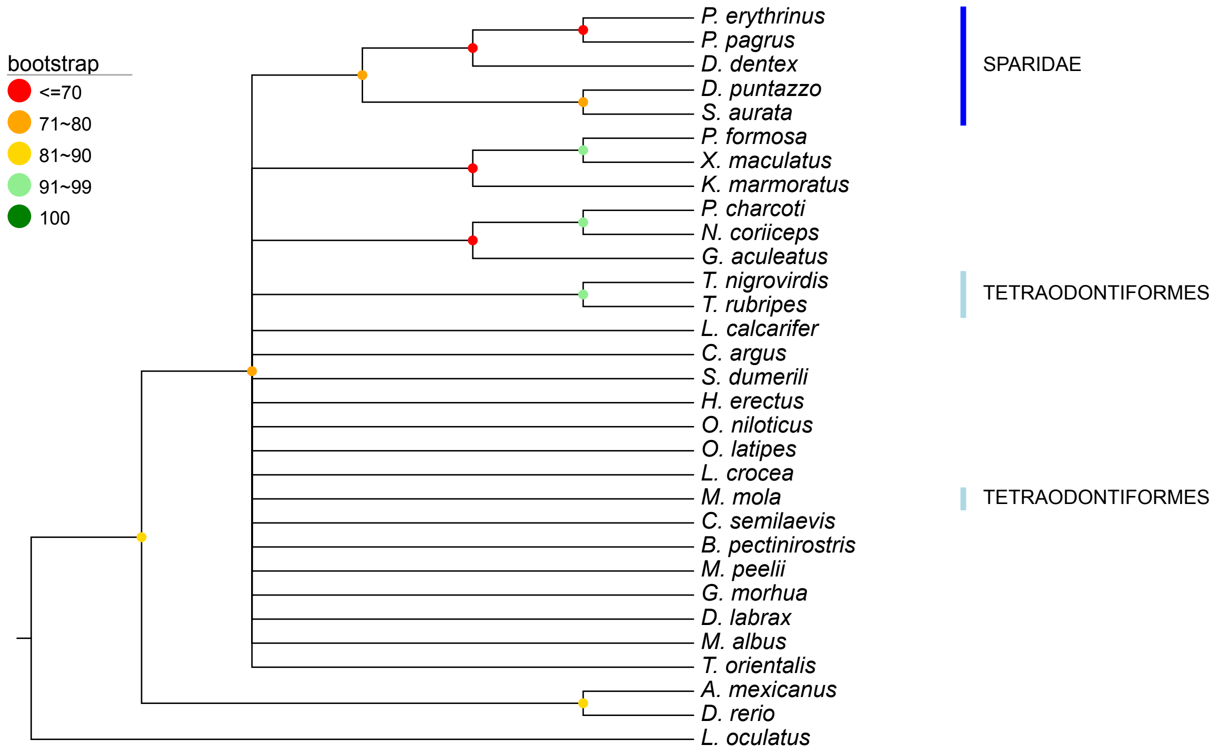

Supplementary Figure 6B Gene tree analysis consensus trees for: A) 135 OrthoFinder and B) 78 PorthoMCL groups with all 31 species present. Figures C and D show the results of IC/ICA calculation by RAxML, for OrthoFinder and PorthoMCL respectively

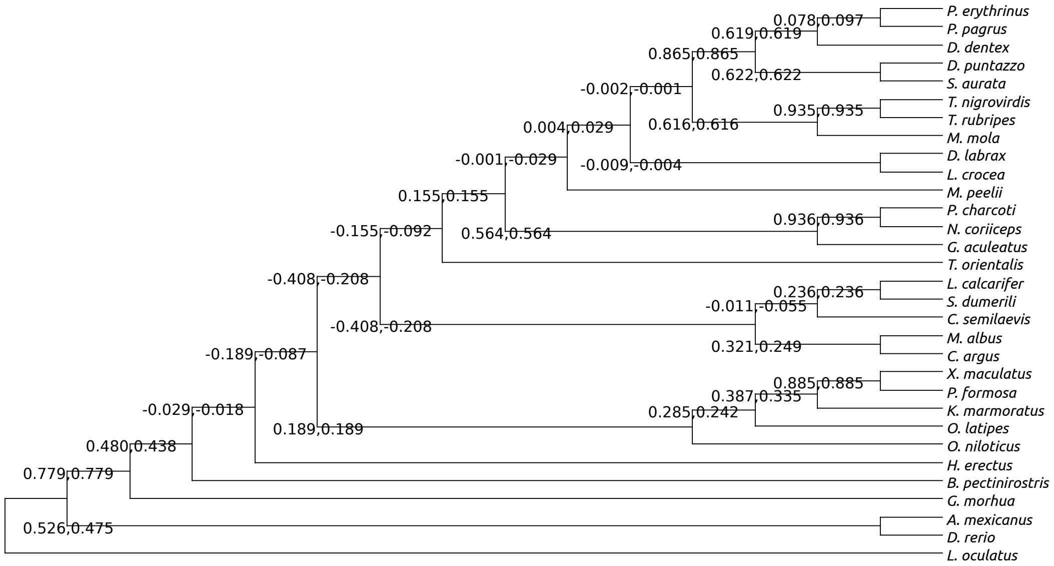

Supplementary Figure 6C Gene tree analysis consensus trees for: A) 135 OrthoFinder and B) 78 PorthoMCL groups with all 31 species present. Figures C and D show the results of IC/ICA calculation by RAxML, for OrthoFinder and PorthoMCL respectively

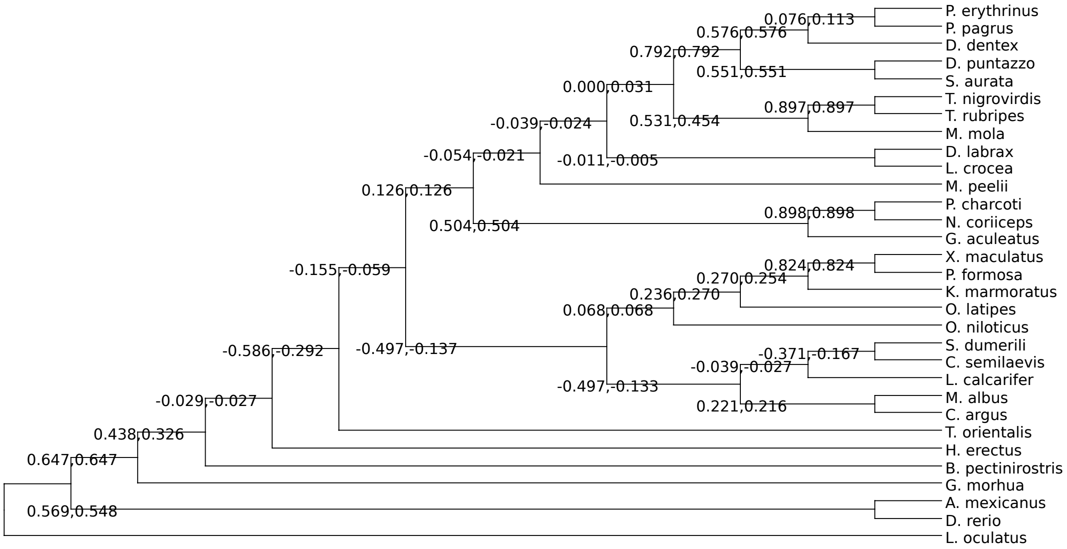

Supplementary Figure 6D Gene tree analysis consensus trees for: A) 135 OrthoFinder and B) 78 PorthoMCL groups with all 31 species present. Figures C and D show the results of IC/ICA calculation by RAxML, for OrthoFinder and PorthoMCL respectively

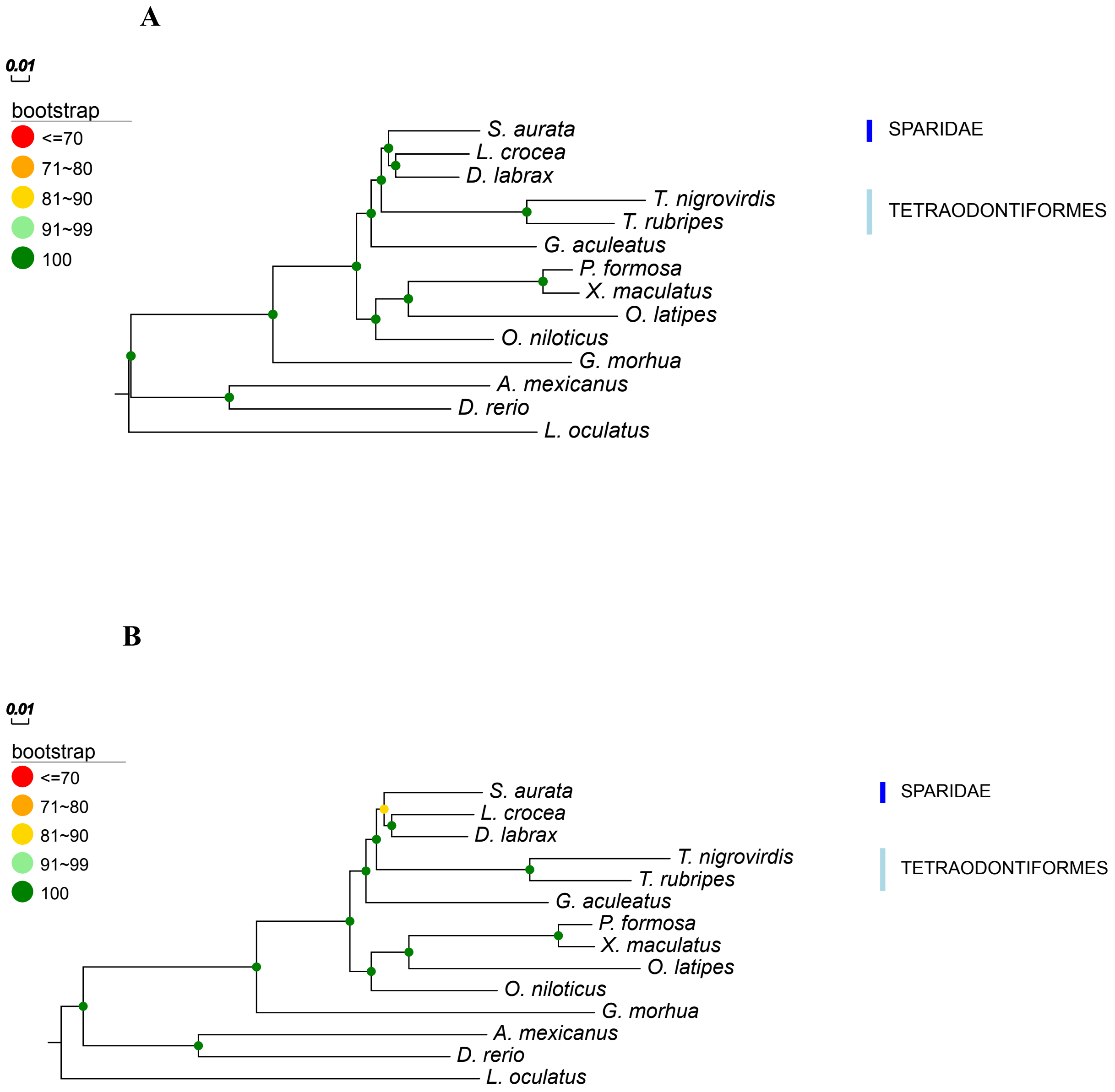

Supplementary Figure 7 Maximum likelihood trees with the 14 species used in gilthead seabream genome paper for: A) 2,192 OrthoFinder and B) 1,366 PorthoMCL single-copy groups with at least 13 species present.

SUPPLEMENTARY TABLES

Supplementary table 1. Quality assessment of the 31 proteomes using BUSCO. The 5 Sparidae gene-sets are depicted with bold characters. The actinopterygii-specific library of BUSCO contains 4,584 annotated genes. The *D. puntazzo* dataset contained the smallest amount of BUSCO-annotated genes (3,347).

**Species**	**Complete (single-copy)**	**Duplicated**	**Fragmented**	**Missing**
A. mexicanus	4,285 (4,062)	223	238	61
B. pectinirostris	4,339 (3,987)	352	70	175
C. argus	4,202 (4,052)	150	214	168
C. semilaevis	4,300 (3,512)	788	58	226
D. rerio	4,451 (4,164)	287	87	46
D. dentex	**3,876 (2,536)**	**1,340**	**218**	**490**
D. labrax	4,441 (4,302)	139	94	49
D. puntazzo	**3,347 (2,274)**	**1,073**	**650**	**587**
G. morhua	4,101 (3,971)	130	413	70
G. aculeatus	4,466 (4,267)	199	92	26

H. erectus	3,688 (3,499)	189	302	594
K. marmoratus	4,449 (3,775)	674	60	75
L. crocea	4,411 (3,674)	737	71	102
L. calcarifer	3,848 (3,622)	226	423	313
L. oculatus	4,360 (4,203)	157	156	68
M. peelii	4,095 (3,941)	154	318	171
M. mola	4,194 (4,043)	151	246	144
M. albus	4,183 (3,335)	848	73	328
N. coriiceps	3,568 (3,124)	444	751	265
O. niloticus	4,531 (4,331)	200	30	23
O. latipes	4,304 (4,127)	177	182	98
P. erythrinus	**3,954 (2,533)**	**1,421**	**210**	**420**
P. pagrus	**3,945 (2,538)**	**1,407**	**152**	**487**
P. charcoti	3,552 (3,432)	120	651	381
P. formosa	4,529 (4,267)	262	45	10
S. dumerili	4,486 (3,943)	543	22	76
S. aurata	**3,910 (2,004)**	**1,906**	**428**	**246**
T. rubripes	4,440 (4,065)	375	105	39

T. nigrovirdis	4,231 (4,049)	182	257	96
T. orientalis	3,762 (3,614)	148	571	251
X. maculatus	4,475 (4,353)	122	94	15
