# Supplementary material for: Phylogenomics investigation of sparids (Teleostei: Spariformes) using high-quality proteomes highlights the importance of taxon sampling
Source: Commun Biol. 2019 Nov 1;2:400. doi: 10.1038/s42003-019-0654-5 (PMC6825128; doi:10.1038/s42003-019-0654-5)
Supplement: Supplementary file 2 — Reporting Summary [file 42003_2019_654_MOESM2_ESM.pdf]

# nature research

Corresponding author(s): Dr Tereza Manousaki

Last updated by author(s): Sep 3, 2019

# Reporting Summary

Nature Research wishes to improve the reproducibility of the work that we publish. This form provides structure for consistency and transparency in reporting. For further information on Nature Research policies, see Authors & Referees and the Editorial Policy Checklist.

## Statistics

For all statistical analyses, confirm that the following items are present in the figure legend, table legend, main text, or Methods section.

| n/a | Confirmed | |
|---|---|---|
| ☐ | ☒ | The exact sample size (*n*) for each experimental group/condition, given as a discrete number and unit of measurement |
| ☐ | ☒ | A statement on whether measurements were taken from distinct samples or whether the same sample was measured repeatedly |
| ☐ | ☒ | The statistical test(s) used AND whether they are one- or two-sided<br>*Only common tests should be described solely by name; describe more complex techniques in the Methods section.* |
| ☒ | ☐ | A description of all covariates tested |
| ☒ | ☐ | A description of any assumptions or corrections, such as tests of normality and adjustment for multiple comparisons |
| ☐ | ☒ | A full description of the statistical parameters including central tendency (e.g. means) or other basic estimates (e.g. regression coefficient) AND variation (e.g. standard deviation) or associated estimates of uncertainty (e.g. confidence intervals) |
| ☐ | ☒ | For null hypothesis testing, the test statistic (e.g. *F*, *t*, *r*) with confidence intervals, effect sizes, degrees of freedom and *P* value noted<br>*Give P values as exact values whenever suitable.* |
| ☐ | ☒ | For Bayesian analysis, information on the choice of priors and Markov chain Monte Carlo settings |
| ☒ | ☐ | For hierarchical and complex designs, identification of the appropriate level for tests and full reporting of outcomes |
| ☒ | ☐ | Estimates of effect sizes (e.g. Cohen's *d*, Pearson's *r*), indicating how they were calculated |

*Our web collection on statistics for biologists contains articles on many of the points above.*

## Software and code

Policy information about availability of computer code

| | |
|---|---|
| Data collection | Publicly available data used in this study were manually retrieved from NCBI, Ensembl and GigaDB databases |
| Data analysis | Open-source tools used in this study: EMBOSS v6.6.0.0, BUSCO v3, OrthoFinder v2.1.2, PorthoMCL, mafft v7, RAxML v8.2.9, ExaBayes v1.4.1, RogueNaRok v1.0, CONSEL v0.20, BioEdit, AliView. Custom python scripts used in this study are deposited in https://github.com/pnatsi/Sparidae_2019 and https://github.com/pnatsi/PorthoMCL-parser |

For manuscripts utilizing custom algorithms or software that are central to the research but not yet described in published literature, software must be made available to editors/reviewers. We strongly encourage code deposition in a community repository (e.g. GitHub). See the Nature Research guidelines for submitting code & software for further information.

## Data

Policy information about availability of data

All manuscripts must include a data availability statement. This statement should provide the following information, where applicable:

- Accession codes, unique identifiers, or web links for publicly available datasets
- A list of figures that have associated raw data
- A description of any restrictions on data availability

The four Sparidae transcriptomes analysed in the present study are available from the corresponding author upon request. Accession numbers for the Sparidae raw sequence reads are: PRJNA395994 (red porgy and common pandora), PRJNA241484 (sharpsnout sea bream) and PRJNA481721 (common dentex, TBU). For the gilthead sea bream, we used the predicted gene-set from its genome paper (Pauletto et al., 2018). The genome of gilthead seabream can be accessed through a dedicated genome browser (http://biocluster.her.hcmr.gr/myGenomeBrowser?portalname=Saurata_v1). All proteomes, orthogroups and alignments used in this study can be found under https://doi.org/10.5281/zenodo.3250770 and scripts used in this study are available in https://github.com/pnatsi/Sparidae_2019 and https://github.com/pnatsi/PorthoMCL-parser

# Field-specific reporting

Please select the one below that is the best fit for your research. If you are not sure, read the appropriate sections before making your selection.

☒ Life sciences ☐ Behavioural & social sciences ☐ Ecological, evolutionary & environmental sciences

For a reference copy of the document with all sections, see nature.com/documents/nr-reporting-summary-flat.pdf

# Life sciences study design

All studies must disclose on these points even when the disclosure is negative.

| | |
|---|---|
| Sample size | No sample-size calculation was performed, the number of species to include was based on genome assembly statistics and coverage across the teleost order spectrum |
| Data exclusions | From the 4 Sparidae transcriptomes, we kept only one isoform per gene, keeping only the longest of the available isoforms |
| Replication | The database from which every proteome was retrieved and the reference paper are shown in table 2. All data used in this study are available https://doi.org/10.5281/zenodo.3250770. Scripts are also available in the github links mentioned above. |
| Randomization | N/A |
| Blinding | N/A |

# Reporting for specific materials, systems and methods

We require information from authors about some types of materials, experimental systems and methods used in many studies. Here, indicate whether each material, system or method listed is relevant to your study. If you are not sure if a list item applies to your research, read the appropriate section before selecting a response.

## Materials & experimental systems

| n/a | Involved in the study |
|---|---|
| ☒ | ☐ Antibodies |
| ☒ | ☐ Eukaryotic cell lines |
| ☒ | ☐ Palaeontology |
| ☒ | ☐ Animals and other organisms |
| ☒ | ☐ Human research participants |
| ☒ | ☐ Clinical data |

## Methods

| n/a | Involved in the study |
|---|---|
| ☒ | ☐ ChIP-seq |
| ☒ | ☐ Flow cytometry |
| ☒ | ☐ MRI-based neuroimaging |
